# Integrative analyses reveal biological function and prognostic role of m7G methylation regulators in high-grade glioma

**DOI:** 10.18632/aging.204999

**Published:** 2023-09-06

**Authors:** Xiaoli Li, Yanyan Li, Na Li, Liangfang Shen, Zhanzhan Li

**Affiliations:** 1 Intensive Care Unit, The First Affiliated Hospital of Zhengzhou University, Zhengzhou, Henan 450052, P.R. China; 2 Department of Nursing, Xiangya Hospital, Central South University, Changsha, Hunan 410008, P.R. China; 3 Department of Oncology, Xiangya Hospital, Central South University, Changsha, Hunan 410008, P.R. China; 4 National Clinical Research Center for Geriatric Disorders, Xiangya Hospital, Central South University, Changsha, Hunan 410008, P.R. China

**Keywords:** m7G methylation, high-grade glioma, immune infiltration, prognosis

## Abstract

Based on 29 m7G regulators, glioma patients were categorized into three groups using data from the Chinese Glioma Genome Atlas (CGGA) and The Cancer Genome Atlas (TCGA) datasets. Distinct characteristics were observed in immune cell infiltration, functional enrichment, and clinical prognosis for every glioma subtype. Analyzing the differentially expressed genes (DEGs) confirmed the distinction among the three m7G clusters. A predictive tool for overall survival (OS) in high-grade glioma patients was developed and confirmed, consisting of 13 m7G regulators forming a prognostic signature. Elevated m7G levels were found to be associated with increased tumor mutation burden and immune activation, indicating a tumor microenvironment characterized by inflammation and a lower overall survival rate. In contrast, reduced m7G scores were linked to a deficiency in immune infiltration, a low burden of mutations, and a non-inflamed phenotype, suggesting a more positive clinical outlook. Additionally, the m7G risk scores were found to impact chemotherapy sensitivity. The m7G predictive pattern shows potential as a marker for the overall survival of patients with high-grade glioma. By significantly improving our comprehension of the functional role of m7G regulators in the advancement of glioma and their impact on clinical results, this study offers valuable perspectives for precision therapy in the management of high-grade glioma.

## INTRODUCTION

Neurosurgery frequently encounters intracranial glioma as the prevailing malignant tumor of primary origin [1]. The prognosis and survival of patients are significantly inversely correlated with the degree of malignancy [2]. This negative correlation is obviously in primary high-grade glioma (Grade III-IV) patients [3]. Nevertheless, the management of glioma remains highly restricted given the present state of technology, and a total recovery is still unattainable. The treatment approach for glioma primarily involves surgical intervention, with radiation therapy and chemotherapy serving as adjunctive treatments [4]. Nevertheless, as a result of the distinctive positioning and invasive growth attributes of brain gliomas, the treatment results frequently prove to be dissatisfactory [4]. Glioma, with its high rates of recurrence and mortality, poses a significant challenge in terms of prognosis. Certainly, an enhanced comprehension of the cause and development of glioma would aid in the prevention and advancement of innovative targeted therapies, ultimately enhancing the prognosis of patients [5].

The m7G gene is responsible for producing the enzyme N7-methylguanosine methyltransferase (N7-MTase), which has a vital function in RNA molecules. The formation of methylated guanosine (m7G) in RNA is catalyzed by N7-MTase [6]. Methylated guanosine, a type of RNA modification, is crucial for preserving RNA stability, contributing to protein synthesis, and controlling gene expression in healthy cells. Extensive research has been conducted on the role of the m7G gene in tumors [7]. Studies indicate that the activity of the m7G gene in cancerous cells is strongly linked to the occurrence, progression, and treatment results of tumors [8]. Methylated guanosine formation is crucial for regulating gene expression, with the m7G gene playing a significant part. RNA stability can be affected by the presence of methylated guanosine, which can also hinder protein synthesis and regulate the interaction of transcription factors [9]. Modifications can potentially affect crucial mechanisms in cancerous cells, including growth, infiltration, and spread [10]. Dysregulated tumor cell proliferation and survival may occur due to abnormal expression of the m7G gene. In particular, the excessive expression of the m7G gene could enhance the proliferation and division of cancer cells, while decreasing the rates of programmed cell death, thus facilitating the progression of tumors [11]. Several studies have suggested that the excessive expression of the m7G gene is linked to the resistance of tumors towards therapy [12–15]. Additional investigation into the regulatory mechanisms and roles of the m7G gene could potentially enhance the advancement of innovative approaches for tumor therapy and enhance prognosis. This study extensively investigated the biological role and predictive significance of m7G RNA methylation regulators in advanced glioma, potentially offering novel perspectives for personalized therapy of high-grade glioma.

## MATERIALS AND METHODS

### Patients and samples

The training and validation datasets were provided by the Chinese Glioma Genome Atlas (CGGA, cgga.org.cn, The CGGA database contains clinical and sequencing data of over 2,000 brain tumor samples from Chinese cohorts, and is equipped with a user-friendly web application for data storage and exploration.) and the Cancer Genome Atlas (TCGA, https://portal.gdc.cancer.gov/). Prior to performing further analyses, the normalization of both datasets took place, and samples lacking follow-up or clinical data were eliminated. Gliomas classified as WHO III and IV were of high grade [16]. After conducting a thorough analysis of the literature, we discovered 29 genes responsible for the regulation of m7G RNA methylation (Supplementary Table 1). As the information was obtained from publicly accessible databases, there was no need for ethical approval.

### Bioinformatic analyses

#### Molecular clustering and differentially expression genes

Consensus analysis was performed using the “Consensus Clustering” package to identify potential molecular subtypes [17]. To decrease the data dimension, the utilization of Principal component analysis (PCA) was implemented [18]. DEGs were identified through differential expression analysis using the R software package ‘limma’.

### GSEA and GSVA analyses

GSEA v4.2.3 was utilized to perform the gene set enrichment analysis (GSEA) [19]. Gene-set variation analysis (GSVA) was conducted using the R package called ‘GSVA’ [20].

### Development and verification of a predictive model

To build and validate a model, as well as determine the final number of genes, we employed the LASSO regression, which is known as the least absolute shrinkage and selection operator [21]. Multivariate Cox regression modeling was subsequently conducted. The risk score for each sample was calculated using the following formula: coef1 multiplied by geneExpression1, plus coef (2) multiplied by gene Expression (2), and so on, up to coef (n) multiplied by gene Expression [22, 23]. Based on the median risk score value, the glioma patients were categorized into either a high-risk or low-risk group. Survival curves of these groups were compared using Kaplan-Meier (KM) analyses, and their ability to predict was assessed using a receiver operating characteristic curve (ROC). To establish a risk classification, the utilization of principal component analysis (PCA) was implemented. An individual risk assessment nomograph was generated. Furthermore, a comparison was made between the high-risk and low-risk groups in terms of the tumor microenvironment and levels of immune infiltration. The correlation between signature genes and drug IC50 values was evaluated.

### Statistical analysis

Differences in categorical variables were examined using a Chi-squared test, whereas the *t*-test was employed to compare means. Multiple groups were compared using one-way ANOVA, and Dunnett's method was used for making multiple comparisons. To examine the associations between two continuous variables, a Spearman correlation analysis was conducted. The hazard ratio (HR) and its 95% confidence intervals (CIs) were calculated using both univariate and multivariate Cox regression. Statistical analyses were conducted using R version 4.0.1, and statistical significance was reported with a significance level of *p* < 0.05.

### Data availability statement

The public dataset used in this study can be found in CGGA (http://www.cgga.org.cn/) and TCGA (https://portal.gdc.cancer.gov/).

## RESULTS

### Characteristics at the molecular level of regulators of m7G in high-grade glioma

**Figure 1:**
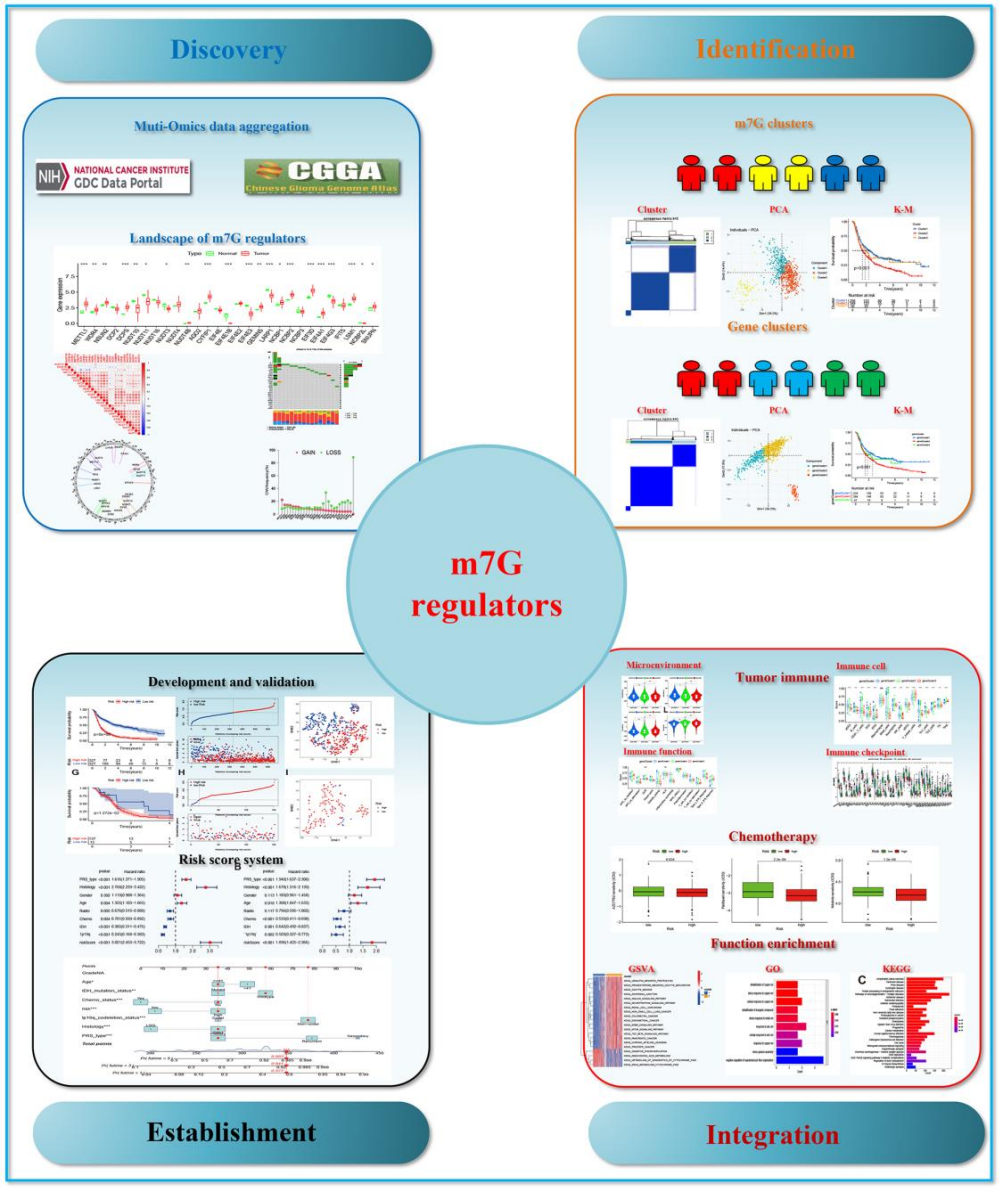
The flow chart of the study.

**Figure 2:**
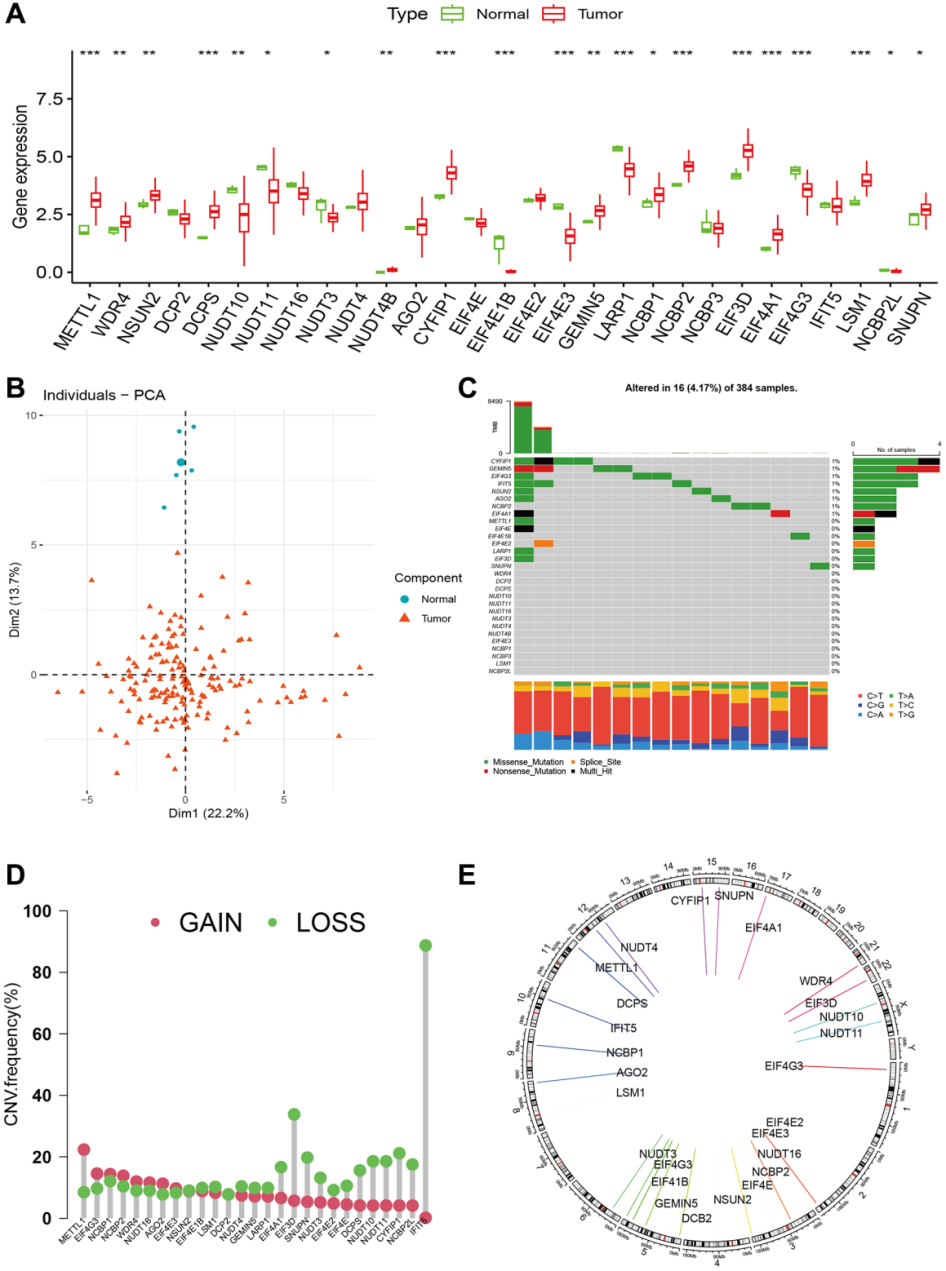
**The landscape of m7G regulators in glioma.** (**A**) Expression levels of m7G regulators between tumor and normal samples. (**B**) PCA of m7G regulators between tumor and normal samples. (**C**) The gene alterations of m7G regulators in advanced glioma. (**D**, **E**) Copy number variation frequencies and chromosomal location of m7G regulators in high-grade glioma.

The entire study flow was depicted in Figure 1. High-grade glioma exhibited high expression levels of twelve genes involved in m7G regulation (METTL1, WDR4, NSUN2, DCPS, NUDT4B, CYFIP1, NCBP1, NCBP2, EIF3D, EIF4A1, LSM1, and SNUPN), while eight genes (NUDT10, NUDT11, NUDT3, EIF4E1B, EIF4E3, LARP1, EIF4G3, and NCBP2L) showed low expression levels. The remaining genes did not display significant differences between tumor and normal samples (Figure 2A, 2B). The levels of gene alterations in m7G genes were below 1%, encompassing CYFIP1, GEMIN5, EIF4G3, IFIT5, NSUN2, AGO2, and NCBP2 (as shown in Figure 2C). The frequency of gain was higher than loss for eight genes, while the frequency of gain for 21 genes was lower (Figure 2D). The distribution of these genes occurred on chromosomes 2, 3, 5, 6, 8, 9, 10, 11, 12, 15, 17, 21, 22, and X can be observed in Figure 2E.

### m7G clusters and their characteristics in high-grade glioma

**Figure 3:**
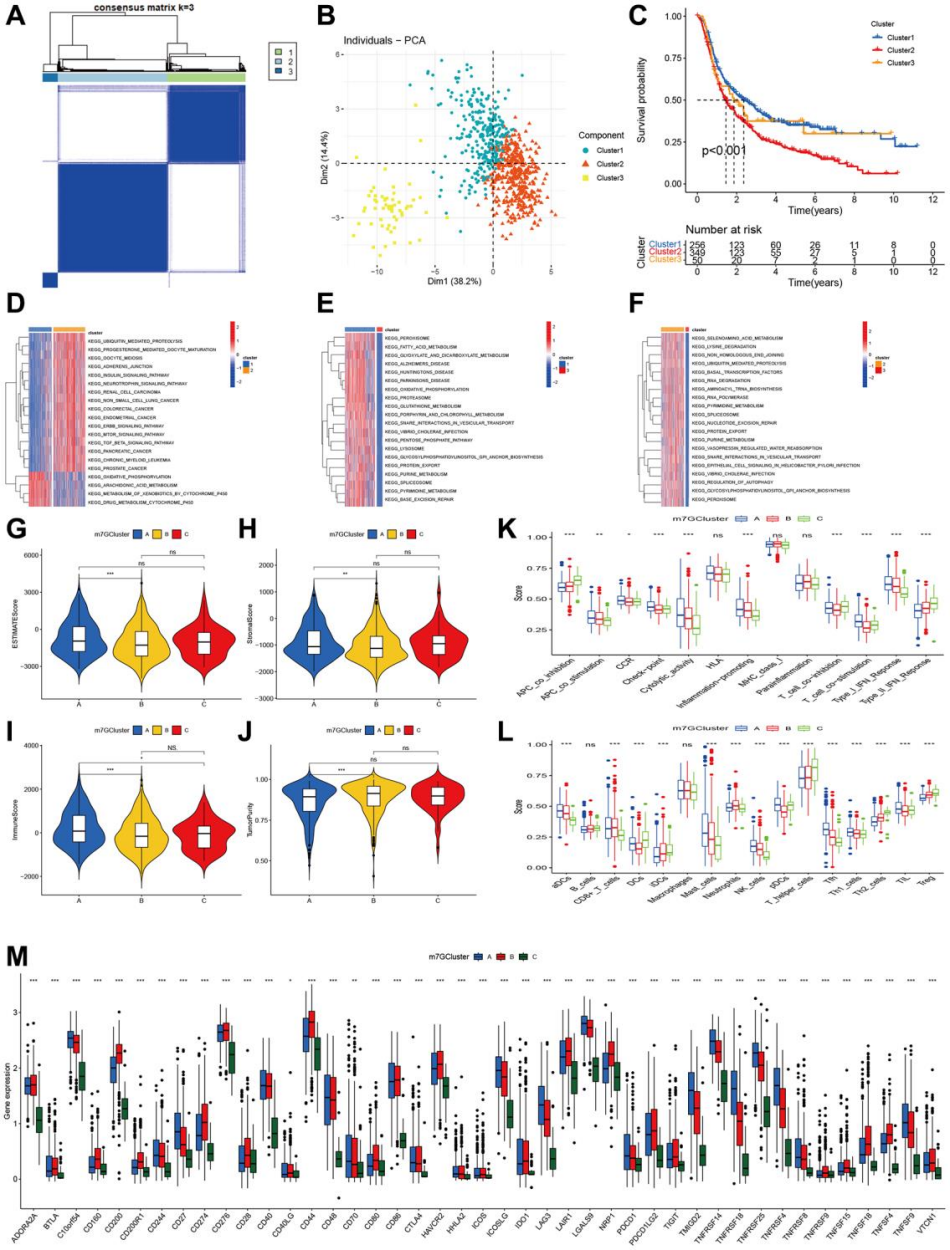
**Clustering of advanced glioma based on m7G regulators.** (**A**) Consensus clustering identified three subtypes. (**B**) PCA showed three distinct clusters. (**C**) Kaplan-Meier analysis indicated significant differences in overall survival among three clusters. (**D**–**F**) GSVA indicated the enriched pathways in three m7G clusters. (**G**–**J**) The differences in ESTIMAT score, stromal score, immune score, and tumor purity across three m7G clusters. (**K**, **L**) Comparisons of immune cell infiltrations level function among three m7G clusters. (**M**) Comparisons of Immune checkpoint-related genes expression among three m7G clusters.

Using 29 m7G genes, we conducted a consensus matrix analysis and acquired three m7G clusters (Cluster 1, Cluster 2, and Cluster 3, as shown in Figure 3A). According to the individual political action committee (PAC), three evident distributions were observed (as shown in Figure 3B). Additionally, the overall survival (OS) displayed notable disparities, as depicted by the Kaplan-Meier curve (Figure 3C). According to the GSVA results, cluster 1 showed significant enrichment in oxidative phosphorylation, arachidonic acid metabolism, xenobiotic metabolism by cytochrome P450, and cytochrome P450 drug metabolism. Cluster 2, on the other hand, exhibited enrichment in ubiquitin-mediated proteolysis, progesterone-mediated oocyte maturation, oocyte meiosis, adherents’ junction, ERBB signaling pathway, and TGF beta signaling pathway. Lastly, cluster 3 demonstrated enrichment in lysosome, GPI anchor biosynthesis, protein export, pyrimidine metabolism, spliceosome, lysosome, and glycolate and dicarboxylate metabolism (Figure 3D–3F).

### Immune status in three m7G clusters

Initially, we assessed the tumor microenvironment in three m7G clusters. Significant variations were observed in the ESTIMATE, stromal, immune scores, and tumor purity. Cluster 1 exhibited elevated ESTIMATE, stromal, and immune scores in comparison to cluster 2 (Figure 3G–3I). Additionally, a similar trend was observed in tumor purity (Figure 3J). Moreover, notable variations in immune function exist among the three clusters. Cluster A exhibited elevated levels of APC co-stimulation, CCR, check-point, cytolytic activity, inflammation-promoting, T cell co-stimulation and inhibition, as well as type I IFN response. Cluster C exhibited greater APC co-inhibition, T cell co-inhibition, and type II IFN response, with cluster B ranking second (Figure 3K). The levels of infiltration by immune cells exhibited a comparable pattern across the three clusters (Figure 3L). Cluster 1 exhibited elevated levels of aDCx, mastocytes, NK cells, pDCs, Tfh, and Th1 cells. Cluster 2 exhibited elevated levels of CD8+ T cells and neutrophils. Cluster 3 exhibited elevated levels of B cells, dendritic cells (DCs), immature dendritic cells (iDCs), T helper cells, Th2 cells, and regulatory T cells (Treg). After assessing the genes related to immune check points, we noticed significant variations among the three clusters for all genes. Cluster 2 exhibited the greatest level of CD274 expression, while cluster 1 followed closely. On the other hand, cluster 3 displayed the lowest expression level. Additionally, the remaining genes demonstrated comparable patterns (Figure 3M).

### Detecting gene clusters by comparing the overlap of differentially expressed genes within m7G clusters

**Figure 4:**
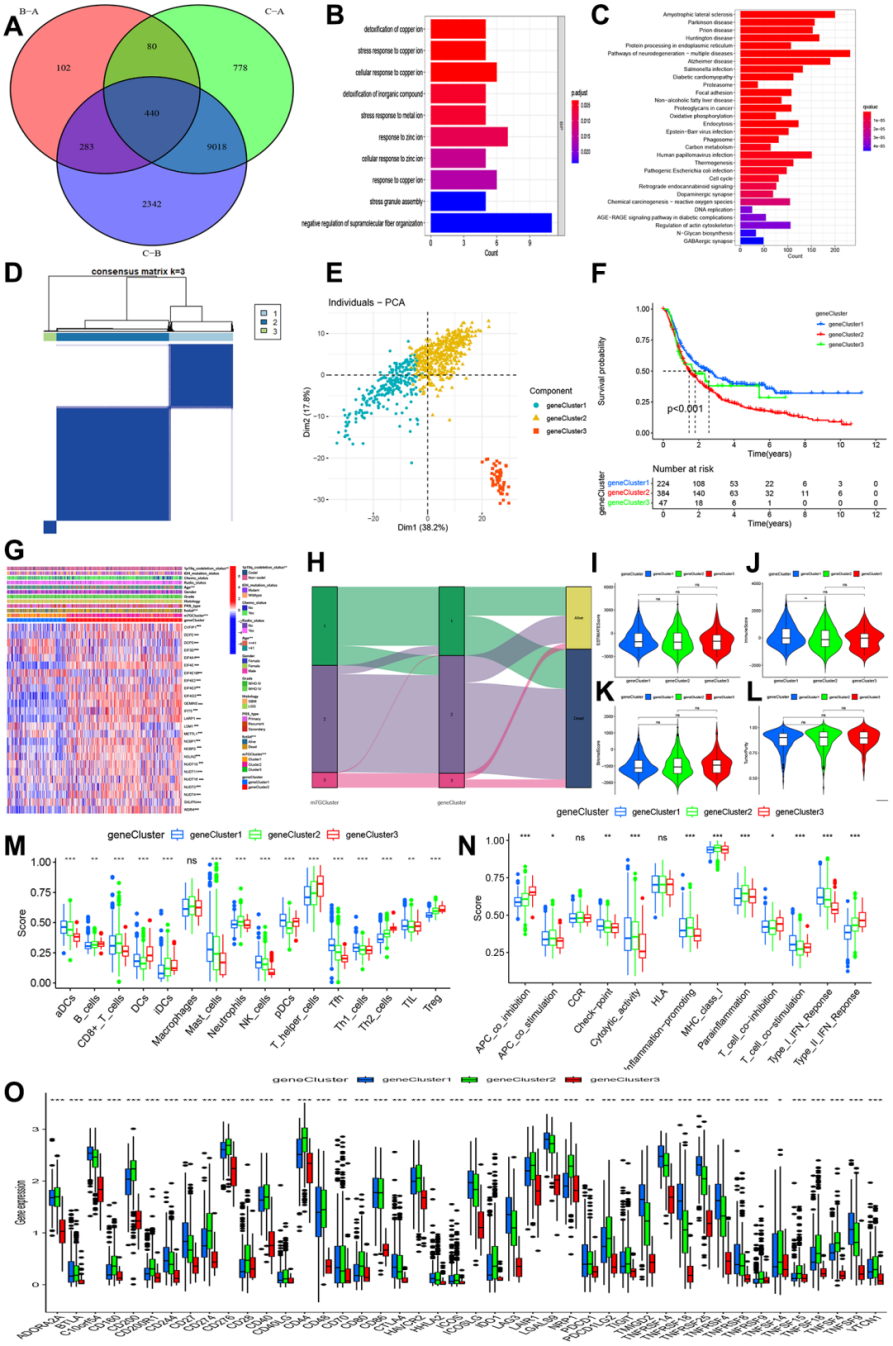
**Identification of gene clusters-based DEGs among three m7G clusters.** (**A**) Venn diagram indicating 440 DEGs among three m7G clusters. (**B**, **C**) GO and KEGG enrichment analyses based on 440 DEGs. (**D**) Consensus matrix identified the number of gene clustering. (**E**) PCA indicated three distinct gene clusters. (**F**) Kaplan-Meier analysis indicated significant differences in overall survival among three gene clusters. (**G**) Correlations of expression profiling, m7G clusters and gene clusters and clinical features. (**H**) Ggalluvial analysis indicated changes from m7G clusters to survival outcomes. (**I**–**L**) Differences in stromal, immune, estimate, and tumor purity. (**M**, **N**) Comparisons of immune cell infiltrations and immune functions. (**O**) Comparisons of immune checkpoint-related genes among three gene clusters.

Among the three m7G clusters, we identified genes that were expressed differently (DGEs), resulting in a total of 440 DGEs (Figure 4A). The GO function enrichment analysis showed that these differentially expressed genes (DGEs) were enriched in the detoxification of copper ions, stress response to copper ions, cellular response to copper ions, response to zinc ions, cellular response to zinc ions, and detoxification of inorganic compounds (Figure 4B). According to the KEGG pathway, these genes were found to be enriched in protein processing in the endoplasmic reticulum, proteasome, focal adhesion, oxidative phosphorylation, phagosome, human papillomavirus infection, and cell cycle, as shown in Figure 4C.

By utilizing the 440 DGEs, we successfully conducted the clustering process and acquired three gene clusters (as depicted in Figure 4D). Figure 4E showed that the PCA revealed three distinct components. According to Figure 4F, the Kaplan-Meier curve showed that gene cluster 2 had the lowest overall survival (OS), followed by gene cluster 3, in comparison to gene cluster 1. Prognosis outcomes, m7G clusters, age, and 1p19q codeletion status exhibited distinct distributions in the clinical characteristics, as depicted in Figure 4G, 4H.

In addition, we assessed the tumor microenvironment and levels of immune infiltration. No significant variations were observed in ESTIMATE, stromal, and tumor purity across gene clusters 1, 2, and 3. However, a notable distinction was found in the immune score, with gene cluster 1 exhibiting a higher immune score compared to gene cluster 2 (Figure 4I–4L). Three gene clusters also exhibited notable variations in immune function and immune cells. Cluster 1 exhibited elevated levels of aDCs, mastocytes, natural killer cells, pDCs, Tfh, Th1 lymphocytes, and tumor-infiltrating lymphocytes. In Figure 4M, gene cluster 2 exhibited elevated levels of CD8+ T cells and neutrophils, while gene cluster 3 showed increased levels of B cells, DCs, iDCs, T helper cells, Th2 cells, and Treg. The highest check-point, cytolytic activity, T cell co-stimulation, and type I IFN response were observed in gene cluster 1, while gene cluster 3 had a slightly lower level, and gene cluster 2 exhibited the lowest immune function. In Figure 4N, gene cluster 3 exhibited the greatest APC co-inhibition, CCR, T cell co-inhibition, and type II IFN response, while gene cluster 2 followed closely behind. On the other hand, gene cluster 1 displayed the lowest levels. All genes exhibited significant differences in the immune check-related gene, as depicted in Figure 4O.

### Creation and verification of a forecasting model relying on m7G regulators

**Figure 5:**
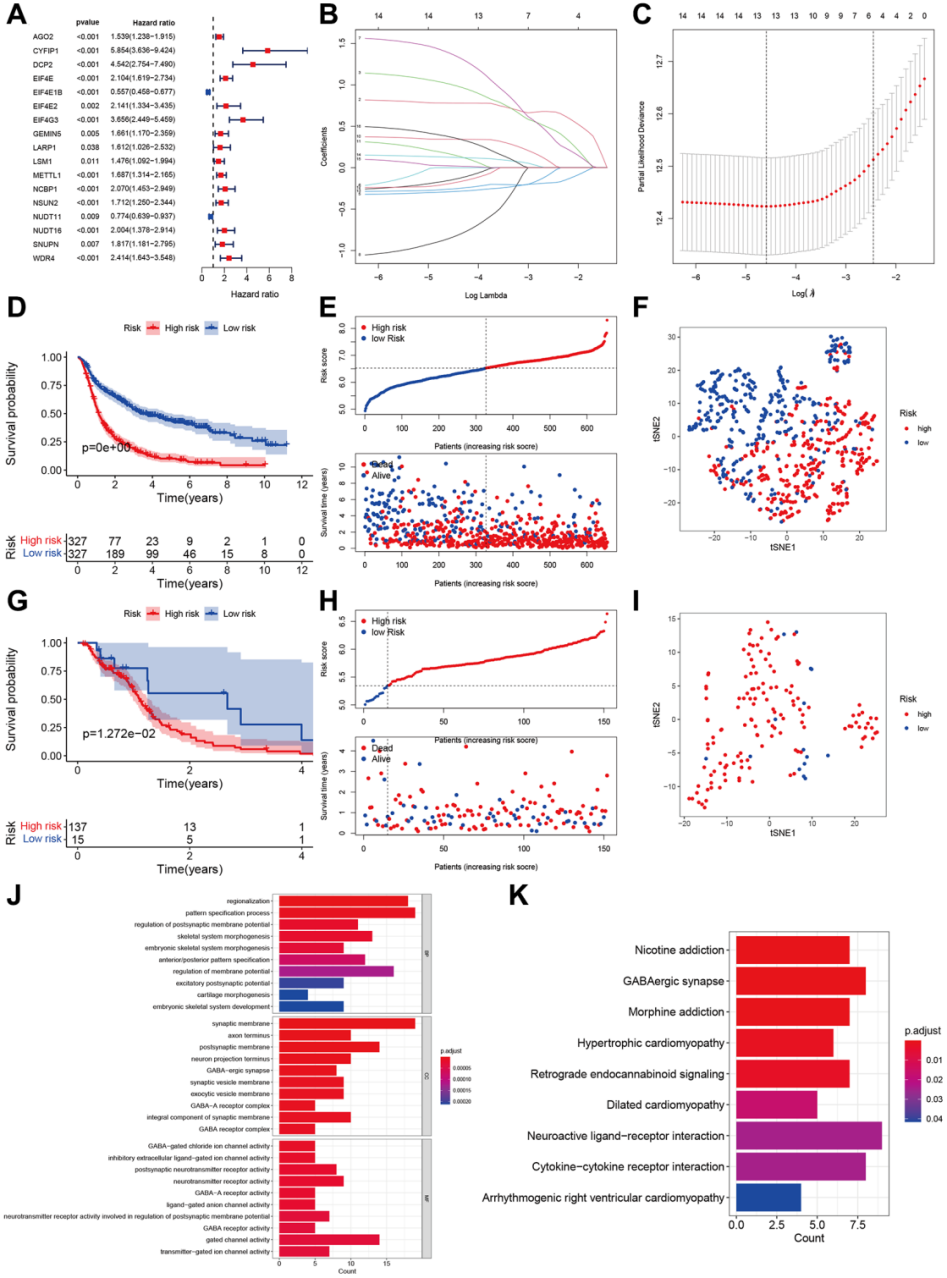
**Development and validation of m7G regulator-based prognostic signatures.** (**A**) Forest plot of univariate cox regression in CGGA. (**B**, **C**) Parameter selection tuning by cross-validation using LASSO regression. (**D**, **E**) Kaplan-Meier curves of risk groups and distribution of risk score and patients in CGGA. (**F**) Display of two components by PCA in CGGA. (**G**) Kaplan-Meier curves of two risk groups and distribution of risk score and patients in TCGA. (**H, I**) Display of two components by PCA in TCGA. (**J**, **K**) GO and KEGG enrichment analyses based on 440 DEGs between high- and low-risk groups.

Initially, we conducted the univariate Cox regression analysis and discovered 17 m7G genes that are linked to prognosis. In high-grade glioma (Figure 5A), EIF4E1B and NUDT11 acted as protective factors for OS, while the remaining factors posed a risk for Spatterwares, we conducted the LASSO regression analysis and discovered 13 genes that were incorporated into the ultimate prognostic model (AGO2, CYFIP1, DCP2, EIF4E1B, EIF4G3, GEMIN5, METTL1, NCBP1, NUDT11, NUDT16, SNUPN, WDR4, LARP1, (Figure 5B, 5C, Supplementary Table 2). Within the CGGA training cohort, we computed the risk score and subsequently categorized the participants into groups of high and low risk. According to the Kaplan-Meier plot, the high-risk category exhibited a less favorable prognosis compared to the low-risk category (Figure 5D, 5E). Additionally, PCA demonstrated distinct distributions for two different individuals (Figure 5F). Significant and comparable findings were also observed in the TCGA validation cohort (Figure 5G–5I).

In order to examine the disparity in function, we acquired the differentially expressed genes (DEGs) between the high-risk and low-risk groups (Supplementary Table 3). The enrichment analysis using the GO function revealed that the high-risk groups showed enrichment in regionalization, regulation of the process that specifies patterns in the postsynaptic membrane potential, GABA-ergic synapse, GABA-A receptor complex, and activity (Figure 5J). Additionally, the KEGG analysis indicated that the high-risk group exhibited enrichment in GABAergic synapse, retrograde endocannabinoid signaling, interaction between neuroactive ligand receptors, and interaction between cytokines and cytokine receptors (Figure 5K).

### The immune landscape and tumor microenvironment of various risk categories

**Figure 6:**
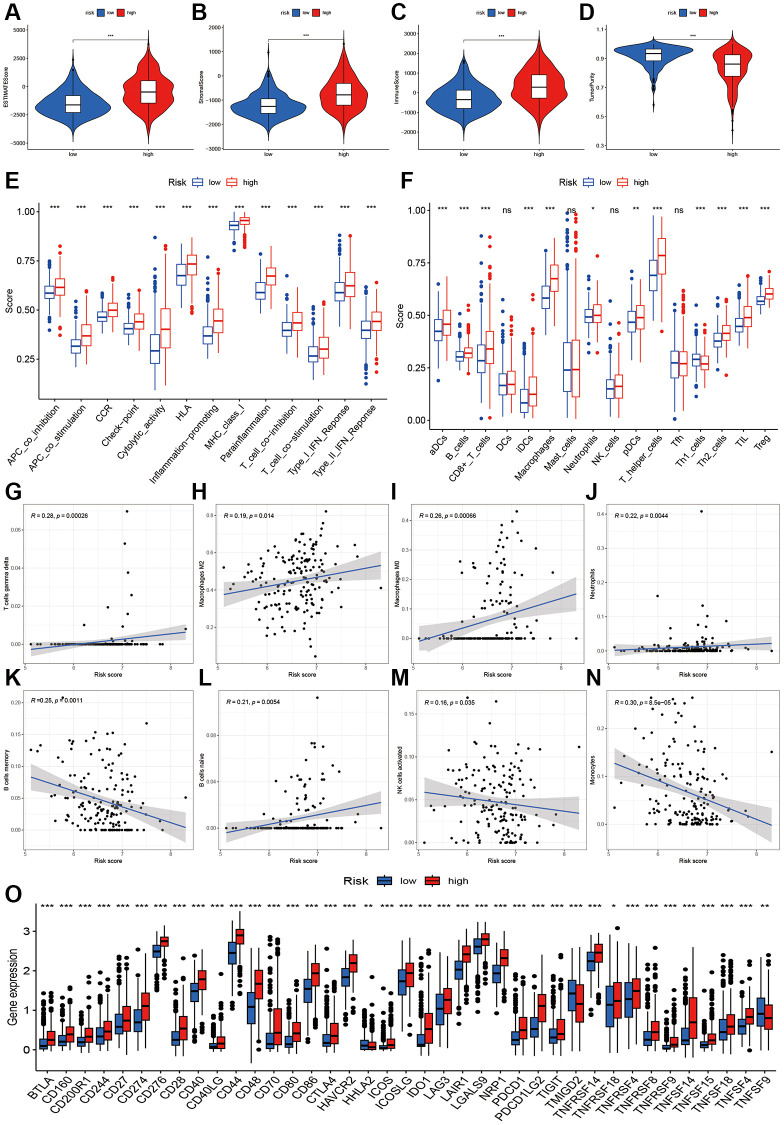
**Immune infiltration analysis of two risk groups.** (**A**–**D**) Boxplot showing the comparative analysis of Estimation, stromal, immune scores, and tumor purity between two risk groups. (**E**, **F**) Comparisons of immune-related cells and functions of two risk groups. (**G**–**N**) Scatter plot showing risk score’s association with regulatory T cells gamma delta, Macrophages M2, M0, Neutrophils, B cells memory and naïve, NK cells activated, and monocytes. (**O**) Comparisons of immune checkpoint-related genes between high- and low-risk groups.

The findings from our study showed that the high-risk group exhibited elevated ESTAMTE score, stromal score, and immune score, whereas the tumor purity was lower in the high-risk group (Figure 6A–6D). These results imply the presence of distinct tumor microenvironments in the high-risk and low-risk groups. In the high-risk group, there was a notable increase in the immune function (APC co-inhibition, APC c0-stimulation, CCR, Check-point, cytolytic activity, HLA, inflammation promoting, MHC class I, Para inflammation, T cell co-inhibition and stimulation, type I and II IFN response, Figure 6E). Likewise, the high-risk group exhibited elevated levels of immune cells such as aDCs, B cells, CD8+T cells, iDCs, macrophages, neutrophils, pDCs, T helper cells, Th1 and Th2 cells, and TIL Treg, compared to the low-risk group (Figure 6F). The correlation analysis showed that the risk score had a positive correlation with T cells gamma delta, M2 and M0 macrophages, and neutrophils (Figure 6G–6J). Conversely, the risk score exhibited an inverse correlation with B cells memory, B cells naïve, NK cell activated, and monocytes (as shown in Figure 6K–6N). The immune-regulated checkpoint genes showed a significant increase in the high-risk category (Figure 6O), suggesting a heightened immune status within this group.

### Independent analysis and risk assessment

**Figure 7:**
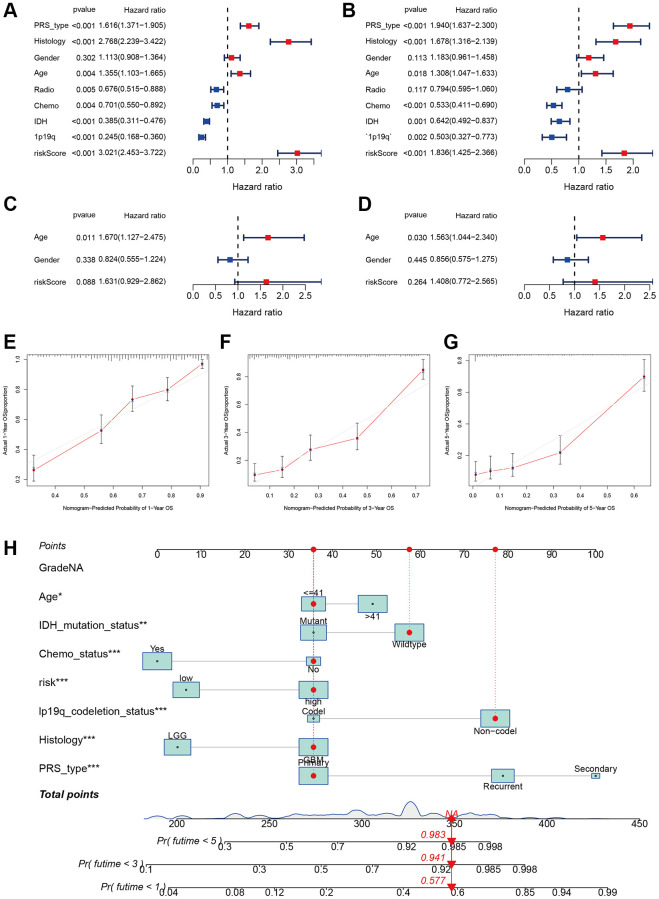
**Independent prognosis analysis of risk score.** (**A**, **B**) Univariate and multivariate cox forest plot of the risk score in CGGA. (**C**, **D**) Univariate and multivariate cox forest plot of the risk score in TCGA. (**E**–**G**) Calibration plots of the nomogram to predict OS over one, three, and five years in the CGGA. (**H**) Nomograph predicting one, three, and five-year OS probabilities as per the m7G prognostic signature.

To confirm the autonomy of the risk score in predicting outcomes, both univariate and multivariate cox regression analyses were conducted. The risk score in the training group showed a significant association with overall survival (univariate hazard ratio (HR) of 3.021, 95% confidence interval (CI) 2.453–3.722, *P* < 0.001; multivariate HR of 1.836, 95% CI 1.425–2.366, *P* < 0.001, as depicted in Figure 7A, 7B). Prognosis in high-grade glioma was also influenced by factors such as PRS categories, histological characteristics, patient age, chemotherapy treatment, presence of IDH mutation, and 1p19q status. Prognosis was not linked to the risk score in the validating group (Figure 7C, 7D). However, the age appears to be a separate prognostic factor in high-grade glioma. Figure 7E–7G displayed a calibration plot showing a precise match between the observed overall survival (OS) and the predicted probability from the nomogram for 1-year, 3-year, and 5-year OS.

Using risk score, age, IDH mutation status, chemotherapy status, 1p19q codeletion status, histology, and PRS type, we developed a personalized risk evaluation tool. According to this tool, the projected overall survival rates for high-grade glioma at 1-year, 3-year, and 5-year were 0.423, 0.069, and 0.017 respectively (Figure 7H).

### Chemotherapy sensitivity

**Figure 8:**
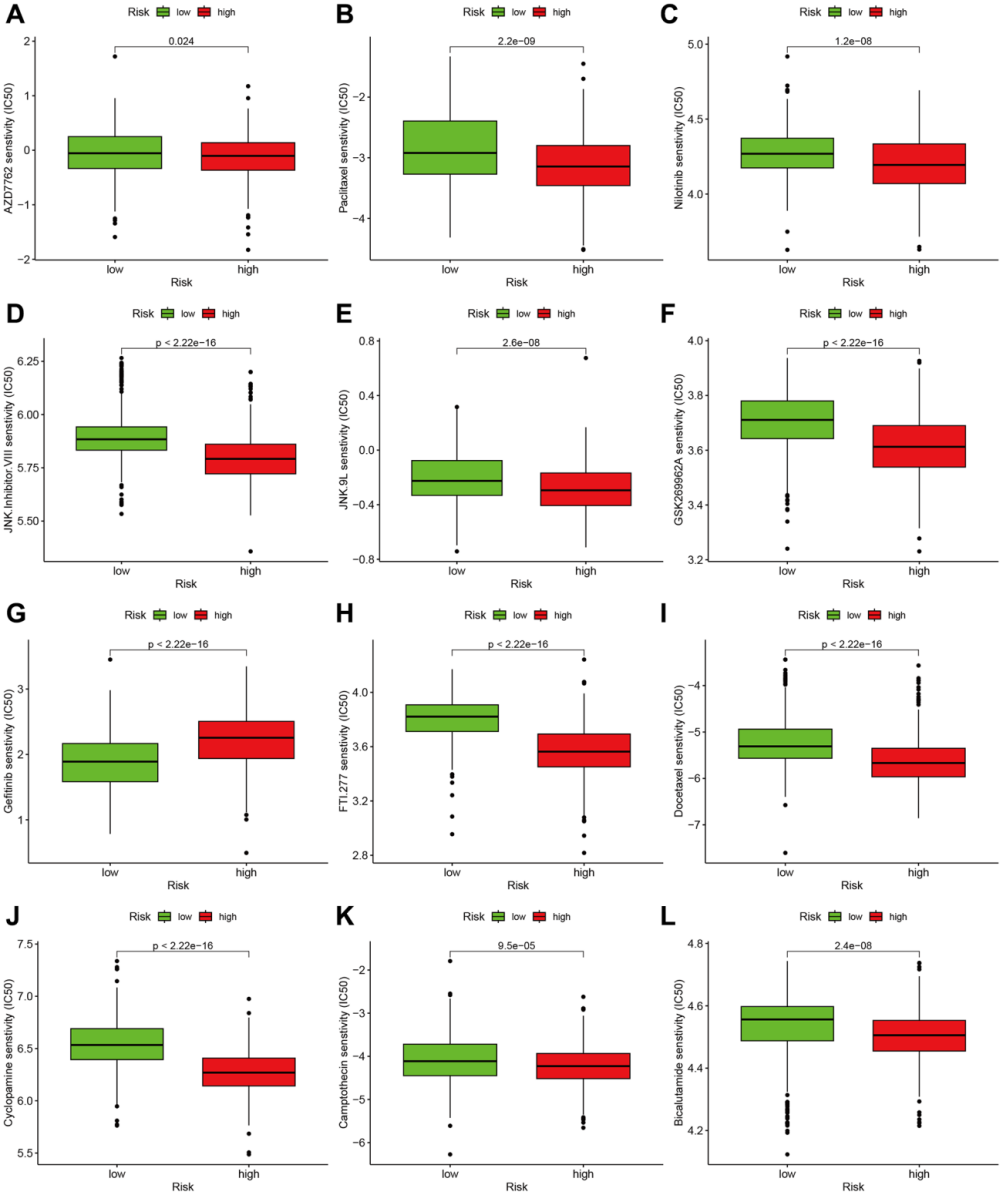
**Effect of m7G risk score on chemotherapy sensitivity.** (**A**–**L**) AZD7762, Paclitaxel, Nilotinib, JNK Inhibitor VIII, JNK.9L, GSK269962A, Gefitinib, FTI.277, Docetaxel, Cyclopamine, Camptothecin, Bicalutamide.

In order to investigate the small molecular compound, we conducted a comparison of the IC50 levels of certain compounds between the high-risk and low-risk groups. In Figure 8A–8L, it was discovered that glioma cells may exhibit resistance to AZD7762, Paclitaxel, Nilotinib, JNK inhibitor VIII, JNK.9L, GSK269962A, Gefitinib, FTI.277, Doctaxel, Cyclopamine, Camptothecin, and Bicalutamide. The other potential compounds could be seen in the Supplementary Table 4.

## DISCUSSION

As per the World Health Organization's classification, gliomas can be categorized into two primary groups: low-grade and high-grade. Grade I or grade II gliomas are primarily categorized as low-grade gliomas, whereas grade III or grade IV glioblastomas are considered high-grade gliomas [24]. The most prevalent primary malignant brain tumors in adults are high-grade gliomas, which encompass anaplastic astrocytoma and glioblastoma multiforme [25]. Glioblastoma multiforme alone constitutes 50% of all gliomas. Diffuse infiltration of the surrounding brain tissue by high-grade gliomas frequently extends across the midline to invade the contralateral brain tissue [26]. Patients have a variety of choices for treatment, including surgical procedures, radiation therapy, and chemotherapy, yet their life expectancy is still relatively limited [27]. Hence, it is imperative to seek out efficient predictive biomarkers and investigate the underlying molecular mechanisms.

We mainly had the following several findings: (1) By utilizing m7 regulators, glioma patients can be classified into three molecular subtypes. These subtypes exhibit distinct biological functions, pathway enrichments, survival prognoses, and immune status. A prognostic model signature was created using 13 m7G regulators, and its accuracy was verified using an independent dataset. The prognostic risk score, obtained from this gene expression pattern, effectively forecasts the likelihood of survival for individuals with glioma. Three sets of genes, derived from differentially expressed genes (DEGs) within the three m7G clusters, exhibit various immune characteristics. Immune infiltration and functioning vary among different risk groups. Discrepancies exist in the enrichment of functions and pathways, as well as in the levels of gene alterations. Several low-mass compounds linked to m7G regulators were detected, potentially providing valuable information on therapeutic approaches. Our research offers new perspectives on the biological processes and immunotherapy for glioma.

The process of m7G RNA methylation entails the addition of a methyl group to the seventh nitrogen (N7) position of the guanine (G) base in RNA, facilitated by methyltransferase enzymes [28]. This alteration has been observed in different RNA molecules, such as the mRNA 5′ cap formation, the middle of mRNA, primary microRNA (pri-miRNA), transfer RNA (tRNA), and ribosomal RNA (rRNA). Methylation of m7G RNA has a regulatory function in various cellular processes, encompassing mRNA transcription, miRNA generation and operation, tRNA durability, and the processing and maturation of 18S rRNA [28]. Following the identification of the m6A modification, m7G RNA methylation has emerged as a relatively new and important focus of study in the realm of epigenetic transcriptomics. In the past few years, increasing proof has emphasized the strong connection between modifications in post-transcriptional methylation and the development of tumors. Nevertheless, the knowledge regarding the characteristics of M7G-altered mRNA in gliomas and its possible contribution to drug resistance remains limited. Additional research is necessary to clarify the influence of m7G RNA methylation on gliomas and its significance for therapeutic strategies [29]. The investigation focused on the analysis of m7G regulators' expression levels to determine the molecular subtypes of glioma. After our investigation, it was discovered that high-grade glioma can be categorized into three separate groups with notably diverse expression patterns and clinical features. Furthermore, distinct subcategories exhibited contrasting clinical outcomes, suggesting the possible effectiveness of m7G regulators in molecular grouping of glioma, thereby implying the potential application of m7G genes in molecular clustering of high-grade glioma. Afterwards, we developed a prognostic model based on m7G that can forecast the overall survival of high-grade glioma. This model was then tested and confirmed using a separate dataset.

Microglia can express some macrophage-related surface markers, including MHC antigens. Therefore, microglia in brain tissue can function as antigen-presenting cells (APCs) 2 [30]. Furthermore, even with the blood-brain barrier in place, activated T cells can penetrate the central nervous system. Most glioma tissues exhibit tumor-infiltrating lymphocytes (TILs), which often indicate a better prognosis for patients. Revealed tumor-specific antigen lymphocytes, indicating that gliomas have a relatively mature acquired immune response *in vitro* cultivation of TILs [31]. Gliomas exhibit specific deficiencies in immune responsiveness due to the following factors: (1) Tumor expansion induces an upsurge in regulatory T cells, impairing APC function and opposing T cell-driven immune reactions. Glioma cells release substances like transforming growth factor-B (TGF-B) and interleukin-10 (IL-10) that hinder the immune response [32]. The proliferative activity of T cells in peripheral blood lymphocytes from glioma patients is reduced when stimulated with T cell mitogens, possibly due to the release of TGF-13 and IL-10. Due to these immune reactivity deficiencies, glioma patients struggle to generate a successful immune reaction against the tumor, consequently enabling the tumor’s uninterrupted growth. In our battle against tumors, it has become crucial to improve the specific response of the immune system to glioma cells using diverse methods as a significant therapeutic strategy [33]. According to our research, it was found that m7G genes may have a correlation with the infiltration of the immune system in high-grade glioma. Cluster 1 exhibited greater ESTIMATE, stromal score, and immune score compared to cluster 2. The status of immune cells and immune function differed. Furthermore, the genes associated with immune checkpoints exhibited varying levels of expression. The presence of antibodies against PD1/PDL1 can strengthen the ability of tumor-infiltrating lymphocytes to kill cancer cells by counteracting the interference of immune functions caused by PD-L1 on the surfaces of cancer cells [34, 35]. Moreover, the PD1/PDL1 signaling pathway diminishes the susceptibility of tumor cells to T cell-induced cell death, suggesting the extensive occurrence of PDL1 in tumor cells [36]. Some clues may be provided by the variations in immune status among the three clusters.

Patients with glioma were categorized into high- and low-risk categories based on their median risk scores, which uncovered notable differences in levels of immune infiltration. Individuals in the more vulnerable category displayed increased stromal, immune, and estimated scores in comparison to those in the less vulnerable category. There was a positive correlation between the risk score and M0 macrophages, resting NK cells, activated CD4 memory T cells, and regulatory T cells. Furthermore, an analysis of gene expression related to immune checkpoints in various risk groups demonstrated a noticeable inclination towards higher expression in individuals with elevated risk.

This study possesses some limitations. Initially, although the training and validation datasets utilized a substantial sample size, it is necessary to obtain supplementary external cohort data in order to verify the precision and consistency of the developed model. Moreover, the TCGA dataset solely contained a restricted set of clinical parameters, and the incorporation of supplementary clinical attributes could potentially influence the results. Lastly, the further experiments are necessary. The development and progression of high-grade glioma are extremely complex, encompassing numerous regulatory pathways and networks. Therefore, these discoveries necessitate additional verification via further *in vivo* and *in vitro* experiments.

In summary, this research presents a unique scientific exploration into the expression trends of m7G controllers in high-grade glioma and their influence on survival. The potential of the m7G prognostic signature to function as a biomarker for the overall survival of patients with high-grade glioma and its potential implications for immunotherapy. Through the modulation of immune responses, extensive analysis suggests that m7G regulators contribute to the advancement of glioma. The results of this research enhance comprehension regarding the participation of m7G regulators in the advancement of high-grade glioma and their impact on medical results, illuminating the possibility of precise therapy in the treatment of high-grade glioma.

## Supplementary Materials

Supplementary Tables 1 and 2

Supplementary Table 3

Supplementary Table 4
