# Supplementary material for: Integrative analyses reveal biological function and prognostic role of m7G methylation regulators in high-grade glioma
Source: Aging (Albany NY). 2023 Sep 6;15(17):8782–99. doi: 10.18632/aging.204999 (PMC10522370; doi:10.18632/aging.204999)
Supplement: Supplementary Tables 1 and 2 [file aging-15-204999-s001.pdf]

SUPPLEMENTARY TABLES

Supplementary Table 1. The 29 m7G genes used for classification.

Genesymbol	Entrez Gene ID	Description
METTL1	4234	Methyltransferase 1
WDR4	10785	WD Repeat Domain 4
NSUN2	54888	NOP2/Sun RNA Methyltransferase 2
DCP2	167227	Decapping MRNA 2
DCPS	28960	Decapping Enzyme, Scavenger
NUDT10	170685	Nudix Hydrolase 10
NUDT11	55190	Nudix Hydrolase 11
NUDT16	131870	Nudix Hydrolase 16
NUDT3	11165	Nudix Hydrolase 3
NUDT4	11163	Nudix Hydrolase 4
NUDT4B	440672	Nudix Hydrolase 4B
AGO2	27161	Argonaute RISC Catalytic Component 2
CYFIP1	23191	Cytoplasmic FMR1 Interacting Protein 1
EIF4E	1977	Eukaryotic Translation Initiation Factor 4E
EIF4E1B	253314	Eukaryotic Translation Initiation Factor 4E Family Member 1B
EIF4E2	9470	Eukaryotic Translation Initiation Factor 4E Family Member 2
EIF4E3	317649	Eukaryotic Translation Initiation Factor 4E Family Member 3
GEMIN5	25929	Gem Nuclear Organelle Associated Protein 5
LARP1	23367	La Ribonucleoprotein 1
NCBP1	4686	Nuclear Cap Binding Protein Subunit 1
NCBP2	22916	Nuclear Cap Binding Protein Subunit 2
NCBP3	55421	Nuclear Cap Binding Protein Subunit 3
EIF3D	8664	Eukaryotic Translation Initiation Factor 3 Subunit D
EIF4A1	1973	Eukaryotic Translation Initiation Factor 4A1
EIF4G3	8672	Eukaryotic Translation Initiation Factor 4 Gamma 3
IFIT5	24138	Interferon Induced Protein with Tetratricopeptide Repeats 5
LSM1	27257	LSM1 Homolog
NCBP2L	392517	Nuclear Cap Binding Protein Subunit 2 Like
SNUPN	10073	Snurportin 1

Supplementary Table 2. 13 identified m7G signature genes in prognostic model.

Gene	Coef	HR
AGO2	−0.153769169	0.8574699
CYFIP1	0.779436234	2.1802428
DCP2	1.011340434	2.7492838
EIF4E1B	−0.291125099	0.7474222
EIF4G3	1.386559685	4.0010614
GEMIN5	−0.855099724	0.4252408
METTL1	0.342305441	1.4081904
NCBP1	0.167862761	1.1827743
NUDT11	−0.259625133	0.7713407

NUDT16	0.126875855	1.1352761
SNUPN	0.022629193	1.0228872
WDR4	0.374922952	1.4548793
LARP1	−0.193499115	0.8240706
